# Cytoskeletal Vimentin Directs Cell‐Cell Transmission of Hepatitis C Virus

**DOI:** 10.1002/advs.202408917

**Published:** 2024-11-29

**Authors:** Yifan Xing, Zeyu Wen, Jie Mei, Xinyi Huang, Shuangshuang Zhao, Jin Zhong, Yaming Jiu

**Affiliations:** ^1^ University of Chinese Academy of Sciences Yuquan Road No. 19(A) Shijingshan District Beijing 100049 P. R. China; ^2^ Key Laboratory of Molecular Virology and Immunology Shanghai Institute of Immunity and Infection Chinese Academy of Sciences Shanghai 200031 P. R. China

**Keywords:** cell surface vimentin, cytoskeletal vimentin, hepatitis C virus, intermediate filaments, viral cell–cell transmission

## Abstract

Hepatitis C virus (HCV) is a major human pathogen causing liver diseases. Although direct‐acting antiviral agents effectively inhibit HCV infection, cell–cell transmission remains a critical venue for HCV persistence in vivo. However, the underlying mechanism of how HCV spreads intercellularly remains elusive. Here, we demonstrated that vimentin, a host intermediate filaments protein, is dispensable for HCV infection in cell models but essential for simulated in vivo infection in differentiated hepatocytes. Genetic removal of vimentin markedly and specifically disrupts HCV cell–cell transmission without influencing cell‐free infection. Through mutual co‐immunoprecipitation screening, we identified that the N‐terminal 1–95 amino acids of vimentin exclusively interact with the HCV envelope protein E1. Introducing either full‐length or head region of vimentin is capable of restoring the cell–cell transmission deficiency in vimentin‐knockout cells. Moreover, we showed that it is vimentin on the plasma membrane of recipient cells that orchestrates HCV cell–cell transmission. Consequently, vimentin antibody, either applied individually or in combination with HCV neutralizing antibody, exerts pronounced inhibition of HCV cell–cell transmission. Together, the results unveil an unrecognized function of vimentin as a unique venue dominating viral transmission, providing novel insights into propelling advancements in vimentin‐targeted anti‐HCV therapies.

## Graphical abstract

A unique role of vimentin in orchestrating HCV cell–cell transmission is characterized, by specifically interacting with HCV structural protein E1 through the head domain of vimentin. It is further proved that vimentin functions through its cell surface form and vimentin antibody exerted a pronounced inhibitory effect on the cell–cell transmission of HCV.

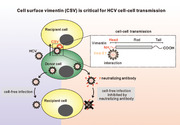

## 1 Introduction

Over the years, accumulating investigations have indicated that viruses spread not only by cell‐free infection but also via cell–cell transmission.^[^ ^1^ ^]^ Compared to cell‐free infection, cell–cell transmission has been proven as an efficient viral strategy to spread within tissues and organs, and can effectively evade host immune responses and neutralize antibody‐based antiviral therapies, thereby contributing to the establishment of persistent virus reservoirs and the pathogenesis of viral infections.^[^ ^2^ ^]^ Mechanistically, virus cell–cell transmission is often associated with biological synapses and cell–cell contacts.^[^ ^1^ ^]^ However, due to limited detection methods and a lack of optimized host targets, studying the mechanisms of viral cell–cell transmission presents a formidable challenge.

Hepatitis C virus (HCV), an enveloped positive‐stranded RNA virus belonging to the Flaviviridae family, is a major cause of liver cirrhosis and hepatocellular carcinoma.^[^ ^3^ ^]^ The 9.6‐kb viral genome encodes a single polyprotein that is cleaved by host and viral proteases into 10 proteins, including three structural proteins (C, E1, and E2) and seven non‐structural proteins (p7, NS2, NS3, NS4A, NS4B, NS5A, and NS5B).^[^ ^4^ ^]^ Among them, E2 is the primary protein that binds to the HCV receptors, hence it is targeted for the design of neutralizing antibodies to prevent the spread of cell‐free viruses.^[^ ^5^ ^]^ In addition to conventional cell‐free infection, HCV has been observed to exploit cell–cell transmission as a mechanism for infecting adjacent cells.^[^ ^5^ ^]^ Vitally, cell–cell transmission contributes to the escape of the host immune response against HCV, leading to viral persistence.^[^ ^2c^ ^]^ It is thus imperative to investigate HCV cell–cell transmission to achieve complete HCV eradication. Previous studies have identified a subset of junction proteins and receptor molecules involved in HCV cell–cell transmission, such as tetraspanin CD81, scavenger receptor class B type I (SR‐BI), claudin‐1 (CLDN1), occluding (OCLN), syndecan (SDC)‐1, SDC‐2, low‐density lipoprotein receptor (LDLR), T cell immunoglobulin and mucin domain‐containing protein 1 (TIM‐1).^[^ ^5^, ^6^ ^]^ However, these molecules also play equally crucial roles in cell‐free infection. Whether there is a specific host molecule exclusively dedicated to HCV cell–cell transmission remains elusive.

Vimentin, a type III cytoskeletal intermediate filament protein, mainly serves in cell morphology maintenance, organelle organization, cell migration, and signal transduction.^[^ ^7^ ^]^ In addition to its well‐established intracellular cytoskeletal network, vimentin has also been found to associate with the plasma membrane, termed cell‐surface vimentin (CSV).^[^ ^8^ ^]^ In recent years, there has been a burgeoning focus on elucidating the role of intracellular vimentin in bacterial and viral infections.^[^ ^9^ ^]^ However, the function of CSV in infections remains largely unexplored. Few previous studies have pointed out that CSV could serve as a putative receptor or co‐receptor to assist in the infection of Japanese encephalitis virus (JEV), severe acute respiratory syndrome coronavirus (SARS‐CoV), and SARS‐CoV‐2.^[^ ^10^ ^]^ Another study elucidated that CSV functions as a restriction factor dampening the infection of human papillomavirus 16 (HPV16).^[^ ^11^ ^]^ These reported functions of CSV are limited to cell‐free viral entry. Whether CSV contributes to viral cell–cell transmission remains inadequately characterized.

In this study, we demonstrated that vimentin is critical for HCV cell–cell transmission but not cell‐free infection, both in vitro and in a simulated in vivo model. Mechanistically, the interaction between the head domain of vimentin and the viral E1 protein is necessary and sufficient for this intercellular transmission. Notably, the application of vimentin antibody, which directly blocks CSV, significantly inhibits HCV cell–cell transmission. Together, our results not only highlight how HCV cell–cell transmission occurs but also reveal a previously unknown virological function of intermediate filaments vimentin.

## 2 Results

### 2.1 Vimentin is Critical for HCV Infection of Differentiated Hepatocytes

**Figure 1:**
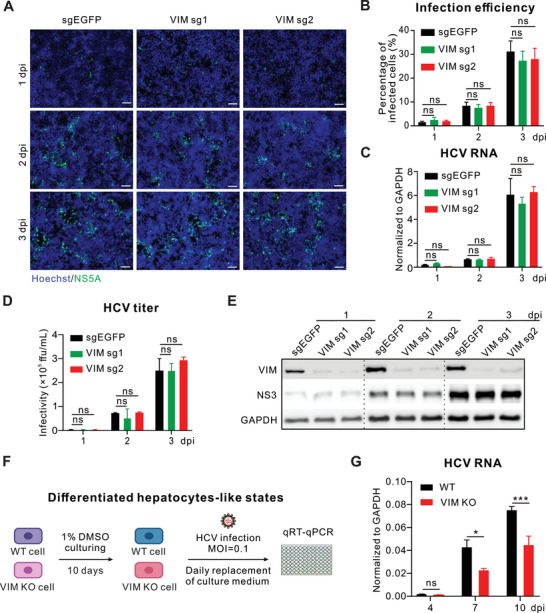
Vimentin is critical for HCV infection of DMSO‐differentiated hepatocytes. A) Cells were infected with HCV (strain JFH1‐GFP) at an MOI of 0.1 for 1, 2, and 3 days, respectively. Cells were then fixed and stained with HCV NS5A antibody (green) and Hoechst 33,258 dye for the nucleus (blue), respectively, to detect infection rates. Scale bars represent 100 µm. B) Statistics of HCV infection rate in control and two VIM KO Huh‐7.5.1 cells. C) Quantification of the intracellular HCV RNA levels in wild‐type and VIM KO cells infected with HCV at an MOI of 0.1 for the indicated time points. D) Quantification of the viral titers in wild type and VIM KO cells were infected with HCV at an MOI of 0.1 for the indicated time points. E) Western blotting verified the level of HCV viral protein NS3 in wild‐type and two VIM KO cell lines. F) Schematic diagram of DMSO‐induced differentiated hepatocytes followed by HCV infection experimental design. G) Time‐dependent analysis of HCV RNA according to experimental design in (F). Data are represented as mean ± SD. Two‐way ANOVA was used for statistical analysis. No significant difference (ns), *p* > 0.05; ^*^ *p* < 0.05; ^***^ *p* < 0.001.

Cytoskeletal vimentin plays an essential role in multiple virus infections.^[^ ^9^, ^12^ ^]^ To explore the potential role of vimentin in the HCV life cycle, we depleted vimentin by the CRISPR/Cas9 method in HCV permissive Huh‐7.5.1 cells with two sgRNA targets (Figure S1A, Supporting Information). Diminished vimentin showed little effect on cell growth compared to wild‐type cells (Figure S1B, Supporting Information). We then conducted time‐course infection experiments with HCV (strain JFH‐1) at an MOI of 0.1, a low MOI that easily amplifies differences during multiple rounds of cell‐free infections. We examined the infection rates (**Figures** **1**A,B), intracellular viral RNA (Figure 1C), extracellular viral titers (Figure 1D), and viral protein production levels (Figure 1E) at 1‐, 2‐ and 3‐ days post‐infection (dpi), respectively. None of these virological indexes were impacted by the removal of vimentin. These data thus indicate that the host cytoskeletal vimentin does not contribute to the HCV life cycle during cell‐free infection.

Given the limitations of the above in vitro cell culture HCV infection model, it may not fully replicate the HCV infection dynamics in vivo. A previous study indicated that human hepatoma‐derived Huh7 cells cultured in the presence of 1% dimethyl sulfoxide (DMSO) turn cytologically differentiated and transition into a nondividing in vivo‐like state, characterized by the induction of hepatocyte‐specific genes.^[^ ^13^ ^]^ We then set up the DMSO‐culture system to investigate the potential function of vimentin under more physiologically relevant conditions (Figure 1F). Briefly, wild‐type and VIM KO cells were cultured in a medium containing 1% DMSO for 10 days to induce differentiation. Subsequently, the cells were infected with HCV at an MOI of 0.1, and the supernatant was replaced daily with fresh medium containing 1% DMSO to maintain cell viability and minimize cell‐free virus‐mediated infection. Different from the conventional cell‐culture infection model, DMSO‐differentiated VIM KO cells displayed a significantly decreased HCV RNA level compared to wild‐type cells, and this disparity was progressively amplified with extended culture duration (Figure 1G). These results demonstrate the important role of vimentin in HCV spreading in differentiated hepatocytes that resemble the in vivo condition.

### 2.2 Vimentin Specifically Orchestrates HCV Cell–Cell Transmission

**Figure 2:**
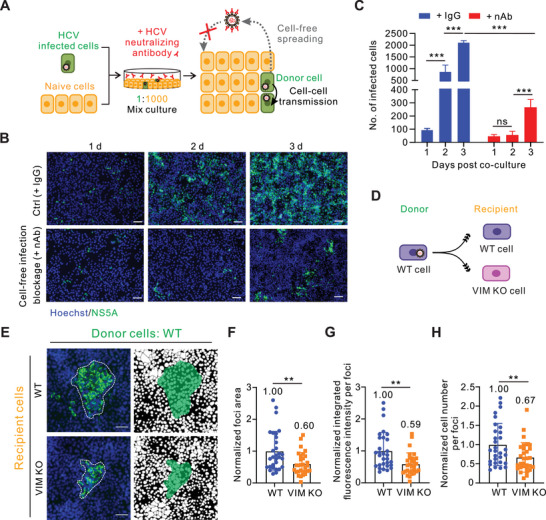
Vimentin specifically orchestrates HCV cell–cell transmission. A) Schematic diagram of cell–cell transmission assay. HCVcc (strain JFH1‐GFP) infected Huh‐7.5.1 cells (donor) mixed with naive Huh‐7.5.1 control cells, vimentin knockout cells, or vimentin rescue cells (recipient) at a ratio of 1:1000, respectively. Cells were then seeded in a plate with 90% density. Neutralizing antibody was added to block cell‐free virus infection. Cells were fixed for immunofluorescence microscopy 3 days after co‐culturing. B) Representative fluorescent images of JFH1‐GFP‐positive cell foci in control (IgG) and cell–cell transmission (HCV nAb) condition. Cells were stained with Hoechst 33 258 dye for nucleus (blue) and HCV NS5A was shown in green, respectively. Scale bars represent 50 µm. C) Quantifications of the number of HCV‐positive cells per focus in (B). D) Schematic diagram of experimental design. E) Representative fluorescent images of HCV‐positive cell foci in the experimental design in (D). The blue channel represents the cell nucleus and the green channel represents the HCV NS5A protein. Mask pictures are used to visualize differences more directly. Scale bars represent 50 µm. F) Quantification of HCV‐positive foci area in the experimental design in (D). G) Quantification of HCV‐positive foci integrated fluorescence intensity in the experimental design in (D). H) Quantification of HCV‐positive cell number per foci in the experimental design in (D). *n* ≥ 25 foci were used for quantification in each group. Data are represented as mean ± SD. Student *t*‐test was used for statistical analysis. No significant difference (ns), *p* > 0.05; ^**^ *p* < 0.01; ^***^ *p* < 0.001.

Given that the DMSO‐differentiated hepatocytes are non‐dividing and in close contact with adjacent cells in which HCV tends to utilize cell–cell transmission for propagation, we hypothesize that vimentin may participate in HCV cell–cell transmission instead of conventional cell‐free infection. To test this hypothesis, we established a system to assess the efficiency of HCV cell–cell transmission by extracellular application of anti‐HCV envelope glycoprotein 2 (E2) neutralizing antibody (nAb) to block HCV cell‐free infection,^[^ ^5^, ^6^ ^]^ thereby allowing for the specific detection of cell–cell transmission. Series concentrations (0.3–6 µg mL^−1^) of HCV E2‐specific monoclonal nAb employed were capable of suppressing HCV cell‐free infection. We then selected a medium concentration of 3 µg mL^−1^ nAb for the subsequent experiments (Figure S2A, Supporting Information). Practically, Huh‐7.5.1 cells that were almost entirely infected with GFP‐tagged HCV (referred to as donor) were mixed with naïve Huh‐7.5.1 cells (referred to as recipient) at a ratio of 1:1000 and seeded to confluence to allow for cell–cell transmission (Figure S2B, Supporting Information). Concurrently, HCV nAb was applied to the culture medium continuously for 3 days (**Figure** **2**A). Expectedly, HCV nAb treatment remarkably decreased the number of infected cells compared to the untreated group (Figures 2B,C). However, HCV nAb could not completely block the HCV infection at late stages, which resulted from cell–cell transmission (Figures 2B,C).

To determine whether vimentin participates in HCV cell–cell transmission, we set up the assay with wild‐type cells as donors and either wild‐type or VIM KO cells as recipients (Figure 2D). The efficiency of cell–cell transmission was measured by quantifying the spread area, the integrated fluorescence intensity, and the cell number of GFP‐positive HCV foci, respectively (Figure S2C, Supporting Information). Intriguingly, the efficiency of HCV cell–cell transmission exhibited a marked reduction in VIM KO cells relative to wild‐type cells when HCV nAb was applied to impede cell‐free infection (Figures 2E–H). Apart from the nAb treatment, carboxymethylcellulose (CMC) sodium medium is another effective means to impede the dissemination of cell‐free virus. Consistently, we observed a compromised reduction in cell–cell transmission in the context of a CMC semi‐solid medium (Figures S2D,E, Supporting Information). Moreover, overexpression of full‐length vimentin in wild‐type cells further enhanced the HCV cell–cell transmission (Figures S2F,G, Supporting Information). Together, these results demonstrate a critical role of vimentin in HCV cell–cell transmission.

**Figure 3:**
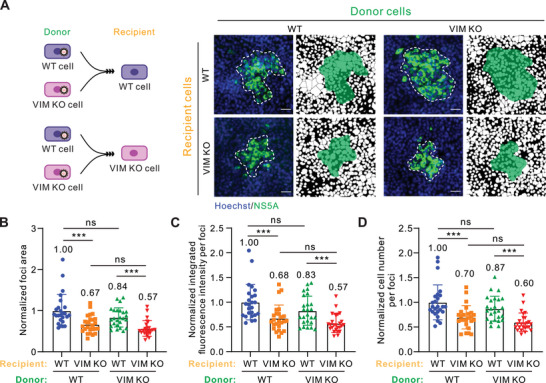
Recipient cell vimentin is critical for HCV cell–cell transmission. A) Schematic diagram of experimental design and representative fluorescent images of HCV‐positive cell foci when using wild‐type or VIM KO cells as donor or recipient. The blue channel represents the cell nucleus and the green channel represents the HCV NS5A protein. Mask pictures are used to visualize differences more directly. Scale bars represent 50 µm. B) Quantification of HCV‐positive foci area in the experimental design in (A). C) Quantification of HCV‐positive foci integrated fluorescence intensity in the experimental design in (A). D) Quantification of HCV‐positive cell number per foci in the experimental design in (A). *n* ≥ 25 foci were used for quantification in each group. Data are represented as mean ± SD. Student *t*‐test was used for statistical analysis. No significant difference (ns), *p* > 0.05; ^***^ *p* < 0.001.

Next, we investigated whether the role of vimentin is exerted by donor or recipient cells (**Figure** **3**A). Cell–cell transmission assay was replicated with permutations and combinations of wild‐type and VIM KO cells as donor and/or recipient cells, respectively. We found that HCV transmission from wild‐type to VIM KO cells showed a ≈50% decrease, which did not exhibit an additive effect in the case of VIM KO to VIM KO cells (Figures 3B–D). Consistently, there was no obvious difference in the transmission efficiency when wild‐type cells acted as recipients regardless of the type of donor cells (either wild‐type or VIM KO) (Figures 3B–D). Together, these data suggested that vimentin in recipient cells plays a crucial role in orchestrating HCV cell–cell transmission.

### 2.3 Vimentin Specifically Interacts with HCV E1 Protein

**Figure 4:**
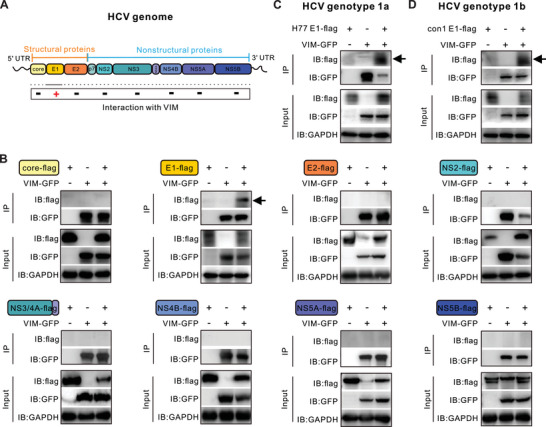
HCV E1 protein interacts with vimentin. A) Schematic diagram of HCV genome encoded proteins. B) Co‐IP assay followed by Western blotting to test the association of vimentin with HCV viral proteins, including E1, E2, core, NS2, NS3/4A, NS4B, NS5A, and NS5B. HEK293T cells were co‐transfected with plasmids expressing vimentin‐GFP together with Flag‐tagged HCV proteins. Cell lysates collected on day 2 post‐transfection were immunoprecipitated with anti‐GFP beads. Anti‐flag antibody was used to detect the interacted viral protein with vimentin. C) Interaction of vimentin‐GFP with flag‐tagged HCV genotype 1a (H77 strain) E1 protein. D) Interaction of vimentin‐GFP with flag‐tagged HCV genotype 1b (con1 strain) E1 protein.

To explore the viral components that potentially interact with vimentin in the process of cell–cell transmission, we constructed the flag‐tagged expression plasmids of three structural proteins (core, E1, and E2) and six nonstructural proteins (NS2, NS3, NS4A, NS4B, NS5A, and NS5B) of HCV (JFH‐1 strain with genotype 2a) (**Figure** **4**A). By co‐transfection of individual viral plasmids with vimentin‐GFP into HEK293T cells, followed by co‐immunoprecipitation, we found that vimentin exclusively interacts with HCV envelope glycoprotein E1, a structural protein surrounding the surface of the assembled viral particles,^[^ ^14^ ^]^ but not with other viral proteins (Figure 4B). Furthermore, we determined that both endogenous vimentin and purified recombinant vimentin protein exhibit an analogous interaction with the transfected flag‐tagged E1 (Figures S3A,B, Supporting Information). To verify the broad spectrum of this interaction, we examined the interaction of vimentin and E1 proteins from two other genotypes of HCV. Likewise, vimentin interacts with H77 (genotype 1a) and con1 (genotype 1b) E1 (Figures 4C,D).

### 2.4 The Head Domain of Vimentin is Sufficient for HCV E1 Interaction and Subsequent Cell–Cell Transmission

**Figure 5:**
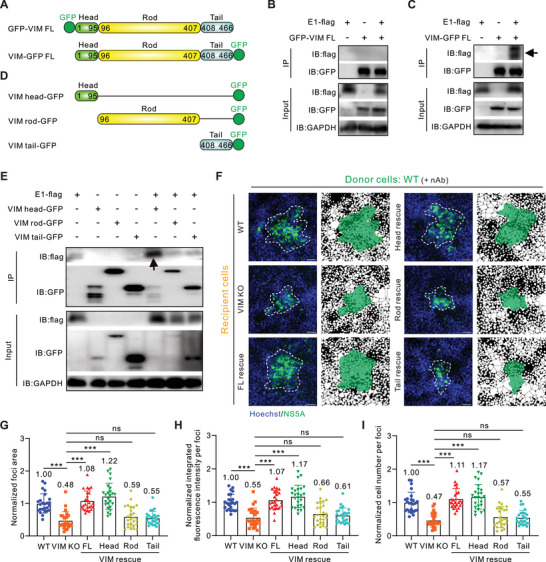
The head domain of vimentin is necessary and sufficient for HCV cell–cell transmission. A) Schematic diagram of N‐terminal GFP‐tagged full‐length vimentin (GFP‐VIM FL) and C‐terminal GFP‐tagged full‐length vimentin (VIM‐GFP FL). B,C) Co‐IP assay followed by Western blotting to test the association of GFP‐VIM FL or VIM‐GFP FL with HCV E1 protein, respectively. HEK293T cells were co‐transfected with plasmids expressing GFP‐VIM FL or VIM‐GFP FL together with Flag‐tagged HCV E1. Cell lysates collected on day 2 post‐transfection were immunoprecipitated with anti‐GFP beads. Anti‐flag antibody was used to detect the co‐immunoprecipitation of E1. D) Schematic diagram of C‐terminal GFP‐tagged vimentin truncations. Vimentin head domain, 1–95 aa; vimentin rod domain, 96–407 aa; vimentin tail domain, 408–466 aa. E) Co‐IP assay followed by Western blotting to test the association of vimentin truncations with HCV E1 protein. HEK293T cells were co‐transfected with plasmids expressing indicated VIM truncations together with Flag‐tagged HCV E1. Cell lysates collected on day 2 post‐transfection were immunoprecipitated with anti‐GFP beads. Anti‐flag antibody was used to detect the co‐immunoprecipitation of E1. F) Representative fluorescent images of HCV‐positive cell foci in different vimentin truncation‐expressed cell lines. The blue channel represents the cell nucleus and the green channel represents the HCV NS5A protein. Mask pictures are used to visualize differences more directly. Scale bars represent 50 µm. G) Quantification of HCV‐positive foci area in fluorescent images in (F). H) Quantification of HCV‐positive foci integrated fluorescence intensity in fluorescent images in (F). I) Quantification of HCV‐positive cell number per foci in fluorescent images in (F). *n* ≥ 25 foci were used for quantification in each group. Data are represented as mean ± SD. Student *t*‐test was used for statistical analysis. No significant difference (ns), *p* > 0.05; ^***^ *p* < 0.001.

To investigate the potential influence of GFP fusion to vimentin on its interaction with HCV E1, we tagged GFP at the N‐ or C‐terminus of vimentin, respectively (**Figure** **5**A). It appears that GFP on the N‐terminus, but not the C‐terminus of vimentin disrupts the interaction (Figures 5B,C), implying the N‐terminus of vimentin may participate in the interaction between E1 protein. Therefore, the C‐terminal tagged GFP vimentin construct was selected for further study.

Vimentin consists of variable non‐helical random coil head and tail domains at the N‐ and C‐terminus, flanking an α‐helical central rod domain.^[^ ^15^ ^]^ To ascertain which domain of vimentin plays a pivotal role in the interaction, we constructed corresponding vimentin truncations, all of which were tagged with GFP at their C‐terminus (Figure 5D). Intriguingly, only the vimentin head domain (1‐95 aa) was identified to interact with HCV E1 (Figure 5E). Next, we ectopically expressed the full‐length and the above three truncated vimentins in VIM KO cells for the subsequent co‐culture experiments (Figure S4A, Supporting Information). Briefly, wild‐type Huh‐7.5.1 cells were used as the donors, while VIM KO cells introduced with different truncations were served as recipients. Interestingly, both the full‐length and the head domain of vimentin are capable of restoring the compromised cell–cell transmission, but not the rod and tail domain of vimentin (Figures 5F–I). Taken together, our results suggested that the head domain of vimentin is critical for its interaction with E1 as well as for HCV cell–cell transmission.

The N‐terminus of vimentin may undergo various post‐translational modifications, among which phosphorylation at the Ser39, Ser56, and Ser83 amino acid residues has a significant impact on the function of vimentin.^[^ ^16^ ^]^ To assess whether phosphorylation of these sites may influence the intercellular transmission of HCV, we first detected the protein level of these phosphorylation sites with specific antibodies and found that there is no difference in HCV‐infected cells compared to naïve cells (Figure S5A, Supporting Information). Next, we overexpressed these mutants and assessed the impact of these mutations on cell–cell transmission. Results showed that none of the three phosphorylation sites is required for the vimentin‐dependent HCV cell–cell transmission (Figures S5B–F, Supporting Information), indicating the dispensable role of these three phosphor sites.

### 2.5 Cell Surface Vimentin is Essential for HCV Cell–Cell Transmission

**Figure 6:**
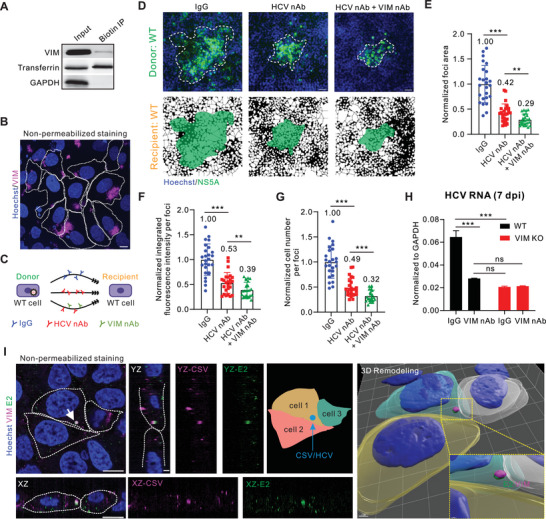
Cell surface vimentin is essential for HCV cell–cell transmission. A) Cell surface protein biotinylation and isolation followed by Western blotting to test the level of cell surface vimentin. B) Huh‐7.5.1 cell no permeation staining. Cells without Triton X‐100 and stained with Hoechst 33 258 dye for nucleus (blue), and anti‐chicken‐vimentin antibodies (magenta), respectively. Cell outlines are depicted by white dashed lines. Scale bars represent 10 µm. C) Schematic diagram of experimental design. D) Representative fluorescent images of HCV‐positive cell foci in the experimental design in (C). The blue channel represents the cell nucleus and the green channel represents the HCV NS5A protein. Mask pictures are used to visualize differences more directly. Scale bars represent 50 µm. E) Quantification of HCV‐positive foci area in the experimental design in (C). F) Quantification of HCV‐positive foci integrated fluorescence intensity in the experimental design in (C). G) Quantification of HCV‐positive cell number per foci in the experimental design in (C). *n* ≥ 25 foci were used for quantification in each group. H) HCV RNA level in the presence of control IgG or vimentin neutralizing antibody in wild‐type and VIM KO cells at 7 days post‐infection in the DMSO system. I) Huh‐7.5.1 cells was stained with Hoechst for nucleus (blue), anti‐chicken‐vimentin antibody (magenta), and anti‐E2 antibody (green), respectively under a non‐permeabilization condition. Cell outlines are depicted by white dashed lines. Scale bars represent 10 µm. The 3D reconstruction of the cell was performed using Imaris 9.5.1 software. Scale bars represent 7 and 0.5 µm, respectively. Data are represented as mean ± SD. Student *t*‐test was used for statistical analysis. No significant difference (ns), *p* > 0.05; ^**^ *p* < 0.01; ^***^ *p* < 0.001.

Our previous results showed that vimentin interacts with HCV surface E1 protein, implying the potential role of vimentin in the transmission of intact HCV particles. In addition, vimentin could be localized on the cell surface referring to CSV.^[^ ^8^ ^]^ We thus hypothesized this interaction may occur on the cell surface. A cell surface biotinylation and isolation experiment followed by anti‐vimentin Western blotting verified the existence of CSV in Huh‐7.5.1 cells (**Figure** **6**A). Moreover, CSV can also be visualized in the hepatoma cells by non‐permeabilized immunofluorescence (Figure 6B). Of note, CSV likely exists at cell edges, which is intuitively aligned with the role of vimentin during HCV transmission.

We presumed that an anti‐vimentin antibody may block CSV and thus inhibit HCV cell–cell transmission. Considering that vimentin does not affect HCV cell‐free infection, we employed a strategy of using anti‐E2 nAb in combination with anti‐vimentin Ab to evaluate their impact on HCV cell–cell transmission, to exclude the influence of cell‐free virus (Figure 6C). Anti‐vimentin Ab combined with anti‐E2 nAb are capable of further reducing the HCV cell–cell transmission efficiency compared to anti‐E2 nAb alone (Figures 6D–G), indicating that CSV could potentially serve as a therapeutic cellular target for inhibiting HCV cell–cell transmission. To further ascertain whether the blockade of CSV exerted an inhibitory effect, we supplemented the cell culture supernatant with anti‐vimentin Ab after HCV infection in the in vivo mimicking DMSO system. The HCV RNA level was reduced by the application of anti‐vimentin Ab in DMSO‐differentiated wild‐type cells but not in VIM KO cells (Figure 6H), confirming the target‐specificity of anti‐vimentin Ab.

To visualize the spatial localization of CSV and HCV particles, we co‐stained CSV and HCV E2 (there is no available anti‐E1 antibody. E1 and E2 always exist in the form of multimers on virions) under non‐permeable conditions. As expected, we observed that HCV‐positive foci and CSV are co‐localized on cell membranes (Figure 6I), supporting our hypothesis that CSV interacts with HCV virions and thus facilitates viral intercellular transmission.

## 3 Discussion

**Figure 7:**
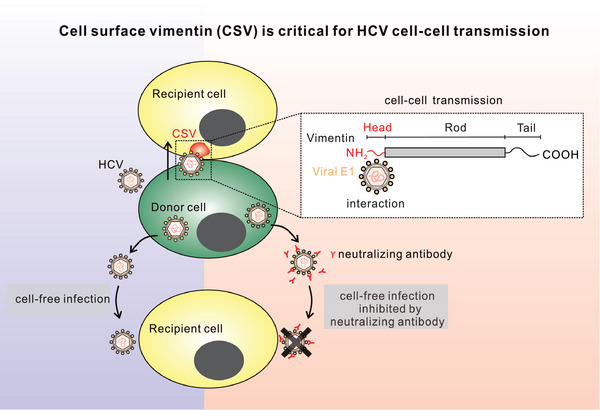
Diagram showing that cell surface vimentin orchestrates HCV cell–cell transmission. While anti‐HCV neutralizing antibodies can inhibit cell‐free HCV infection, cell–cell transmission remains unaffected. Vimentin is not required for cell‐free HCV infection, instead, it contributes to the cell–cell transmission of HCV virions. Specifically, cell surface vimentin of recipient cells interacts with the HCV E1 protein through its head domain. This interaction occurs in the vicinity of cell edges, which could help the attachment of virions to recipient cells, ultimately promoting HCV transmission.

In this study, we discovered an unprecedented function of intermediate filaments vimentin in HCV spread (**Figure** **7**). We revealed that: 1) the head domain of cell surface vimentin (CSV) in recipient cells is required for HCV cell–cell transmission but not for cell‐free infection via interacting with viral E1; 2) vimentin antibody significantly blocks the intercellular transmission of HCV by targeting the viral binding site on the cell surface. Our findings thus lead to a deeper understanding of HCV cell–cell transmission which will contribute to anti‐infection therapy and the elimination of HCV. Furthermore, this study broadens our understanding of the diverse functions of vimentin during viral infection and indicates that vimentin may play a broader spectrum effect in viral cell–cell transmissions beyond HCV, as a unique host factor.

During in vivo infection, viral cell‐free spread may encounter considerable hindrances such as direct antiviral drugs and host immune responses.^[^ ^1b^ ^]^ Growing evidence underscores the significance of HCV intercellular transmission in chronic infection.^[^ ^17^ ^]^ Importantly, viruses have a much higher probability of developing multiple drug resistance when they are transmitted via a cell–cell mode than through a cell‐free mode, suggesting the necessity of inhibiting cell–cell transmission during antiviral therapy.^[^ ^18^ ^]^ However, due to the hepatic cell specificity of HCV infection and the low efficiency of cell–cell transmission in the cell culture model, studying the specific mechanisms of HCV cell–cell transmission presents significant challenges.^[^ ^5^ ^]^ Our previous work has shown that the HCV virion release and cell‐free infection, but not cell–cell transmission, are significantly reduced if the hepatic cells are grown in a culture medium containing galactose, which triggers changes in the cellular metabolism from aerobic glycolysis to oxidative phosphorylation that more closely mimics the glycometabolism state in the non‐dividing hepatocytes in the liver.^[^ ^19^ ^]^ These results suggest that cell–cell transmission may play a much more significant role in HCV spreading in vivo compared to in vitro cell culture infection system. In the present study, we found that combining HCV E2 neutralizing antibodies (which block the interaction of virions with receptors) with vimentin antibodies can further enhance the antiviral effects (Figures 6D–G), suggesting that the exclusive function of vimentin in HCV transmission may hold great significance in its elimination.

Up to now, due to the lack of a completely successful mouse model for HCV infection, we have not been able to test cell–cell transmission in vivo. We thus utilized the DMSO‐differentiated hepatocytes system to mimic hepatocytes in the liver to assess HCV cell–cell transmission (Figure 1F).^[^ ^13^ ^]^ Compared to our initial observations of vimentin depletion on HCV infection (Figures 1A–E), there was a significant reduction in HCV RNA levels in VIM KO cells in the long‐last DMSO system (Figures 1G,6H). This variation may be attributed to three primary factors. First, under typical infection conditions, the abundance of viral particles in the supernatant and sparse cell density predominantly support the cell‐free mode of viral propagation. Conversely, in the DMSO system, characterized by heightened cellular density and cell–cell contact, HCV may tend to exploit cell–cell transmission as a major mode of dissemination. The second reason may be the altered metabolic dynamics. Our recent study proposed that the metabolic state in hepatocytes is critical for HCV to favor its spread through cell–cell transmission in vivo,^[^ ^19^ ^]^ implying that the spread of HCV virions in DMSO may rely more on cell–cell transmission. The third factor is the extended observation period. While cells cultured under standard conditions exhibit maximal activity within 3–5 days, the DMSO‐culturing system supports prolonged cellular viability (14–20 days), allowing for the amplification of differences resulting from cell–cell transmission over an extended timeframe.

By far, cell–cell transmission mainly occurs via two intercellular structures: biological synapses and tunneling nanotubes (TNTs). Biological synapses are types of cell–cell adhesions composed of actin and tubulin between adjacent cells, which usually do not form long‐lasting contacts.^[^ ^1a^ ^]^ Typically, human immunodeficiency virus (HIV) utilizes virological synapses and infectious synapses to spread between the same and/or different cell types.^[^ ^20^ ^]^ TNTs are thin and long channels (50–200 nm in width and up to 100 µm in length) composed exclusively of actin filaments. Influenza virus (IAV),^[^ ^21^ ^]^ HIV,^[^ ^22^ ^]^ herpesviruses,^[^ ^23^ ^]^ and SARS‐CoV‐2,^[^ ^24^ ^]^ have been reported to utilize TNTs for their intercellular spreading. However, vimentin has been identified in neither of the above‐reported structures for cell–cell transmission. We suspect that the selection of cell–cell transmission mode may be dependent on both virus and cells. Our results thus imply that we may discover a previously unreported vimentin‐dependent viral transmission mode, distinct from synapses and TNTs, which may also function in other pathogens.

CSV has been proposed to function as the viral receptor or co‐receptor for the invasion of SARS‐CoV, SARS‐CoV‐2, and JEV.^[^ ^10^ ^]^ The invasion of bacteria, such as *Listeria monocytogenes*, is also mediated by CSV.^[^ ^25^ ^]^ In our study, we visualized CSV in hepatocytes using biotin membrane extraction and non‐permeabilized immunostaining (Figures 6A,B). CSV is mainly found in the vicinity of cell edge regions (Figure 6B). Furthermore, we observed the colocalization of CSV with HCV envelop protein (Figure 6I). We thus speculate that CSV interacts with HCV particles via structural E1 protein, which facilitates the attachment of virions to recipient cells and ultimately promotes HCV transmission. Vitally, the administration of vimentin antibody conspicuously suppressed HCV infection, irrespective of whether it occurred within the cell–cell transmission model or the DMSO system (Figures 6C–H), further revealing the essential role of CSV in orchestrating HCV intercellular dissemination.

There are four previous studies correlating vimentin with HCV. Two studies indicated the interaction between vimentin and HCV core protein; however, they have not detected the role of vimentin in HCV infection.^[^ ^26^ ^]^ Another study discovered that the expression of vimentin in Huh7 cells is associated with the quantity of HCV core protein, which in turn results in increased HCV production by assessing the core protein levels in the culture supernatants during JFH‐1 infection.^[^ ^27^ ^]^ Additionally, another work indicated that vimentin participates in HCV RNA replication in R1b cells using sub‐genome RNA transfection.^[^ ^28^ ^]^ However, all these studies did not select appropriate systems and detection methods, in terms of authentic viral infection. We suspect the possible reason for these distinctions may be attributed to our in‐depth analysis using hepatocellular carcinoma cell lines and the authentic HCV infection.

Up to date, even though most HCV infections can be cured by direct‐acting antiviral agents (DAAs), the problems of resistant mutations caused by anti‐viral drugs and hepatitis pathogenesis persist as a nuisance. Therefore, it is substantially important to understand the elaborate mechanism of HCV intercellular spreading. Our results underscore the significance of vimentin in HCV cell–cell transmission in both the cell culture model and the simulated in vivo model. We also provide new underlying insights for intermediate firmaments vimentin, in terms of virology and cell biology.

## 4 Conclusion

In conclusion, we have characterized the unique role of vimentin in orchestrating HCV cell–cell transmission. Depletion of vimentin resulted in a remarkable decrease in HCV infection in a simulated in vivo infection model. Mechanistically, vimentin specifically interacts with the HCV structural protein E1 through its head domain to promote HCV intercellular transmission. We further demonstrated that vimentin may function through its cell surface form. Importantly, vimentin antibodies exerted a pronounced inhibitory effect on the cell–cell transmission of HCV. Collectively, our study fills a long‐term gap in our knowledge of the cellular function of vimentin, in addition to the conventional viral life cycle, and provides new insight into the mechanism of HCV cell–cell transmission.

## 5 Experimental Section

### Cell Culture

Huh‐7, Huh‐7.5.1, and HEK293T cells were maintained in high glucose Dulbecco's modified Eagle's medium (DMEM) (GIBCO) supplemented with 10% fetal bovine serum (FBS) (LONSA), 100 U penicillin and 100 µg mL^−1^ streptomycin (GIBCO), 10 mm HEPES (GIBCO), 2 mm L‐glutamine (GIBCO) and MEM Non‐Essential Amino Acids Solution (GIBCO) at 37 °C in a humidified atmosphere with 5% CO_2_.

### Virus Preparation and Quantification of Infectivity Titers

The preparation and titration of HCV cell culture (HCVcc) was as described previously.^[^ ^29^ ^]^ HCV stocks were prepared by infection of Huh‐7.5.1 with a JFH‐1‐derived high titer virus D183.^[^ ^30^ ^]^ For viral titer detection, Huh‐7.5.1 cells (1 × 10^4^) cells were seeded in a 96‐well plate and infected with a serially diluted supernatant for 72 h. The cells were fixed with 4% paraformaldehyde and incubated with an antibody against HCV NS5A protein followed by incubation with Alexa Fluor 488–conjugated secondary antibody and Hoechst 33 258. The stained cells were analyzed by fluorescence microscopy and the viral titers were expressed as focus‐forming units per milliliter of supernatants (ffu mL^−1^).

### Plasmids Construction

The coding sequences of human vimentin were amplified by PCR/RT‐PCR from Huh‐7 cell cDNA, using the following primers: vimentin (forward: ATGTCCACCAGGTCCGTG; reverse: TTATTCAAGGTCATCGTGATGCTGAGA). Green fluorescent protein (GFP) was cloned and fused to the N or C terminal of full‐length vimentin as indicated. Vimentin head (1‐95 aa), rod (96‐407 aa), and tail (408‐466 aa) were cloned and fused with GFP protein in the C terminal. Vimentin mutations (S39A, S56A, and S83A) were constructed by homologous recombination. The amplified PCR products were cloned into the pLVX‐IRES‐Neo vector. Plasmids expressing core, NS2, NS3/4A, NS4B, NS5A, and NS5B contained a FLAG tag at the N terminus. The E1‐ and E2‐expressing plasmids contain a signal peptide at the N terminus and a FLAG tag at the C terminus.^[^ ^31^ ^]^ All the constructs were verified by DNA sequencing.

### Quantitative RT‐PCR

These assays were performed as previously described.^[^ ^32^ ^]^ The cells or supernatant were lysed in TRNzol (Tiangen, Beijing, China) and RNA was isolated following the manufacturer's protocol. The cDNA syntheses were using the ReverTra Ace qPCR RT kit (Toyobo). Real‐time PCR was performed using quantitative PCR SYBR Green real‐time PCR Master Mix (Toyobo) and the specific primers targeting different genes (HCV, forward: TCTGCGGAACCGGTGAGTA; reverse: TCAGGCAGTACCACAAGGC; GAPDH, forward: GAAGGTGAAGGTCGGAGTC; reverse: GAAGATGGTGATGGGATTTC). The expression of target genes was normalized to the expression of GAPDH.

### CRISPR/Cas9‐Mediated Knockout

The sequences of the Single guide RNAs (sgRNAs) were: sgEGFP: GAACCGCATCGAGCTGA; VIM‐sg1: ATTGCTGACGTACGTCACGC; VIM‐sg2: CAGGATGTTCGGCGGCCC. The sgRNA sequences were cloned into lentiCRISPRv2 and subjected to lentivirus packaging and transduction. The knockout of genes was validated by Western blotting.

### Lentivirus Packaging and Infection

1.5 × 10^6^ HEK293T cells were transfected with a mix of 2 µg of transfer vector (pLVX‐based constructs), 1.5 µg of psPAX2 (Addgene), and 1 µg of pMD2.G (Addgene) using PEI. 60–72 h post‐transfection, cell supernatants were passed through 0.45 µm sterile filters. After the lentivirus in fection, puromycin was added to the infected cells to enrich the lentiviral transduction.

### Cell Proliferation Assay

Cell proliferation was measured by Cell Counting Kit‐8 (Beyotime, C0040, CN). Briefly, cells were plated in the density of 1 × 10^4^ in100 µL per well in a 96‐well culture plate and then incubated with10 µL of CCK‐8 regent at 37 °C for 0.5–4 h at the indicated time points. Absorbance at 450 nm was then measured with Synergy H1 Hybrid Multi‐Mode Reader (BioTek, US).

### Western Blotting

Cells were harvested by RIPA Lysis Buffer (CW Biotech) containing Protease Inhibitor Cocktail Set III (Calbiochem) and Phosphatase Inhibitor Cocktail Set II (Calbiochem). Protein concentrations were normalized by BCA Protein Assay. Protein samples were added with 5 × SDS‐PAGE Loading Buffer (NCM Bio) and subjected to SDS‐PAGE. Protein samples were resolved by SDS‐PAGE and transferred onto PVDF membranes (Merck Millipore). Membranes were blocked using 5% skimmed milk, probed with primary antibody, and then with horseradish peroxidase (HRP)‐conjugated secondary antibody. The membranes were subjected to chemiluminescent detection. The following primary antibodies were used: vimentin chicken polyclonal antibody (dilution 1:1000, #ab24525, Abcam); HCV NS3 antibody (dilution 1:1000); GAPDH (dilution 1:1000, #10494‐1‐AP, Proteintech); Monoclonal anti‐flag M2 antibody (dilution 1:1000, #F1804, Sigma‐Aldrich); Anti‐transferrin receptor mouse monoclonal antibody (dilution 1:5000, #ab269513, Abcam); Rabbit polyclonal to GFP (dilution 1:1000, #ab290, Abcam); Anti‐His tag mouse monoclonal antibody (5C3) (dilution 1:1000, #ABT2050, Abbkine). The following secondary antibodies were used: Anti‐rabbit IgG, HRP‐linked Antibody (dilution1:5000, # 7074, Cell Signaling); Anti‐mouse IgG, HRP‐linked Antibody (dilution1:5000, # 7076, Cell Signaling); Anti‐chicken IgM, HRP‐linked Antibody (dilution1:5000, #ab 112 813, Abcam).

### Immunofluorescence Microscopy

For cell surface protein staining, cells cultured on glass slides (VWR, #631‐0150) were fixed in a fixed buffer (2% paraformaldehyde (PFA), 0.2% glutaraldehyde and 1 mm MgCl2 in PBS) for 10 min at room temperature (RT). Cells were then blocked in PBS supplemented with 5% bovine serum albumin (BSA) (ABCONE, #A23088). Both primary and fluorescent‐conjugated secondary antibodies were applied to cells in 1% BSA at RT for 45 min. Cells were mounted in Hoechst Fluoromount‐G reagent (SountherBiotech, 0100–20) and imaged using an Olympus spinSR10 Ixplore spinning disk confocal microscope. The following primary antibodies were used: vimentin chicken polyclonal antibody (dilution 1:200, #ab24525, Abcam); HCV E2 antibody (dilution 1:200, custom‐made by Zhong lab). The following secondary antibodies were used: Alexa Fluor 488 goat anti‐chicken IgY (H+L) (dilution 1:1000; #A11039, Invitrogen). The cell membrane was stained by DiD (#D7757, Invitrogen) to determine the cell outlines.

### HCV Cell–Cell Transmission Assay

To detect the efficiency of HCV cell–cell transmission, Huh‐7.5.1 cells that had been infected with a previously reported recombinant HCVcc expressing GFP‐tagged NS5A^[^ ^33^ ^]^ (donor) at an MOI of 3 for 3 days. Subsequently, the infection rate was assessed using immunofluorescence to ensure that nearly all cells had been infected with HCV. The infected donor cells were then mixed with naïve Huh‐7.5.1 cells (recipient) in a ratio of 1:1000. The donor and recipient cells were co‐cultured in the presence or absence of HCV E2‐specific monoclonal nAb 8D6^[^ ^34^ ^]^ for the indicating time points. A polyclonal chicken antibody was used for cell surface vimentin neutralizing (#ab24525, Abcam) at a ratio of 1:200. The spread efficiency was analyzed by counting the number, fluorescence intensity, and area of every GFP‐positive focus.

### Carboxymethylcellulose (CMC) Sodium Medium Assay

The preceding steps of infection and mix culture are the same as cell–cell transmission assay. After cell adhesion, the culture supernatant was removed, and the pre‐sterilized and prepared non‐flowable CMC semi‐solid culture medium (#C4888, Sigma–Aldrich) was implemented to impede the dissemination of viruses in the supernatant. The spread efficiency was analyzed by counting the number of every GFP‐positive focus.

### Co‐Immunoprecipitation Assay

HEK293T cells were co‐transfected with indicated plasmids and lysed in Triton‐×100 buffer containing 50 mm Tris/HCl, pH 7.5, 150 mm NaCl, 0.5% Triton‐×100, with 2% PMSF, and protease inhibitor (Sigma) at 48 h post‐transfection. The cell lysates were centrifuged with 10,000 g at 4 °C for 10 min and the supernatant was collected. 100 µL supernatant was kept for the input detection and the rest of the supernatant with the same protein was incubated with anti‐GFP beads (KT HEALTH, KTSM1301, CN) for 1 h at 4 °C. Beads were washed four times with lysis buffer and eluted with 100 µL SDS loading buffer at 98 °C for 10 min for further Western Blotting analysis.

### Pull‐Down Assay

Briefly, vector or Flag‐E1 plasmid was transfected into 293T cells. After 48 h, cells were collected using PBS buffer and lysed with ultrasonication. The cell lysates were first incubated with anti‐flag agarose beads (#M20018, Abmart) to separate the flag‐E1 protein, and then purified His‐vimentin protein (#10028‐H08B, SinoBiological) were added for incubation. Western blotting was performed to detect the His tag to determine the interaction.

### Biotinylation and Isolation for Cell Surface Vimentin Detection

Pierce Cell Surface Biotinylation and Isolation Kit (#A44390, Thermo Scientific) was used for cell surface biotinylation. One 10 cm dish of cells was biotinylated in 10 mL PBS containing 250 µg mL^−1^ sulfo‐NHS‐SS‐Biotin for 20 min at room temperature. The labeling solution was removed and each dish was washed 2 additional times with ice‐cold TBS. Cells were harvested in ice‐cold TBS using a cell scraper. Cells were centrifuged at 500 × g for 3 min at 4 °C and supernatant was discarded. 500 µL lysis buffer supplemented with protease inhibitor was added to each dish and cells were transfered to a 1.5 mL microcentrifuge, incubate cells with rotation for 30 min at 4 °C. The homogenate was centrifuged at 15000 × g for 5 min at 4 °C and the supernatant was incubated with NeutrAvidin Agarose with rotation for 2 h. The beads were washed 5 times with wash buffer. Finally, the beads were incubated with elution buffer supplemented with 10 mm DTT at RT for 1 h to elute the surface biotinylated proteins for further Western Blotting analysis.

### DMSO Simulation of In Vivo Condition

The assay was performed according to the previously reported.^[^ ^13^ ^]^ In brief, cells were seeded in wells of a plate, followed by continuous induction of cellular state changes using a complete DMEM medium containing 1% DMSO. After culturing for 10 days until cells reached a non‐proliferative state, they were infected with HCV. Media were changed daily to complete DMEM medium containing 1% DMSO to minimize the impact of cell‐free virus post‐infection. Cellular samples were collected for RT‐qPCR analysis at the indicated time points. To assess the effect of vimentin‐neutralizing antibodies, IgG or vimentin‐neutralizing antibodies were added daily during media changes.

### Statistical Analysis

Statistical analysis was performed using GraphPad PRISM 8. For statistical analyses between two groups, two‐tailed, paired Student t‐tests were applied unless otherwise indicated. For testing between more than two groups ordinary two‐way ANOVA with Tukey's multiple comparisons with individual variances computed for each comparison was performed. The *P* value < 0.05 was considered statistically significant. Data are shown as means ± SD of the means.

## Conflict of Interest

The authors declare no conflict of interest.

## Author Contributions

Y.X. carried out the majority of the experiments and the data interpretation. Z.W. and J.M. implemented some infection and immunofluorescence experiments. X.H. and S.Z. optimized the figure layout. Y.J. and J.Z. conceived, supervised the project, and participated in the interpretation of the result. Y.X., J.Z., and Y.J. wrote the manuscript with contributions from all other authors.

## Supporting information



Supporting Information

## Data Availability

The data that support the findings of this study are available on request from the corresponding author. The data are not publicly available due to privacy or ethical restrictions.
