# Supplementary material for: Cytoskeletal Vimentin Directs Cell‐Cell Transmission of Hepatitis C Virus
Source: Adv Sci (Weinh). 2024 Nov 29;12(3):2408917. doi: 10.1002/advs.202408917 (PMC11744697; doi:10.1002/advs.202408917)
Supplement: Supplementary file 1 — Supporting Information [file ADVS-12-2408917-s001.docx]

Supporting Information

Cytoskeletal vimentin directs cell-cell transmission of hepatitis C virus

Yifan Xing, Zeyu Wen, Jie Mei, Xinyi Huang, Shuangshuang Zhao, Jin Zhong*, and Yaming Jiu*


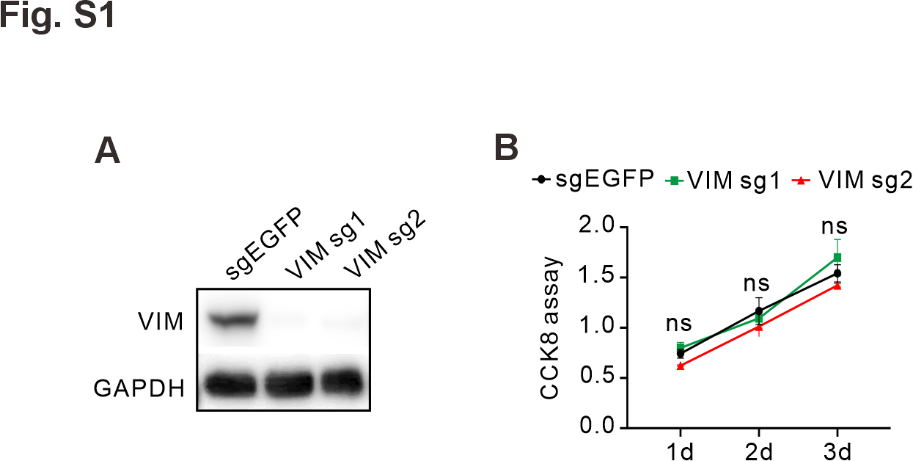


**Figure S1.** Verification of VIM KO. A) Western blotting verified vimentin expression in wild-type and two vimentin knockout cell lines. B) Quantification of the cell proliferation in wild-type and two VIM KO cell lines. No significant difference (ns), p > 0.05.


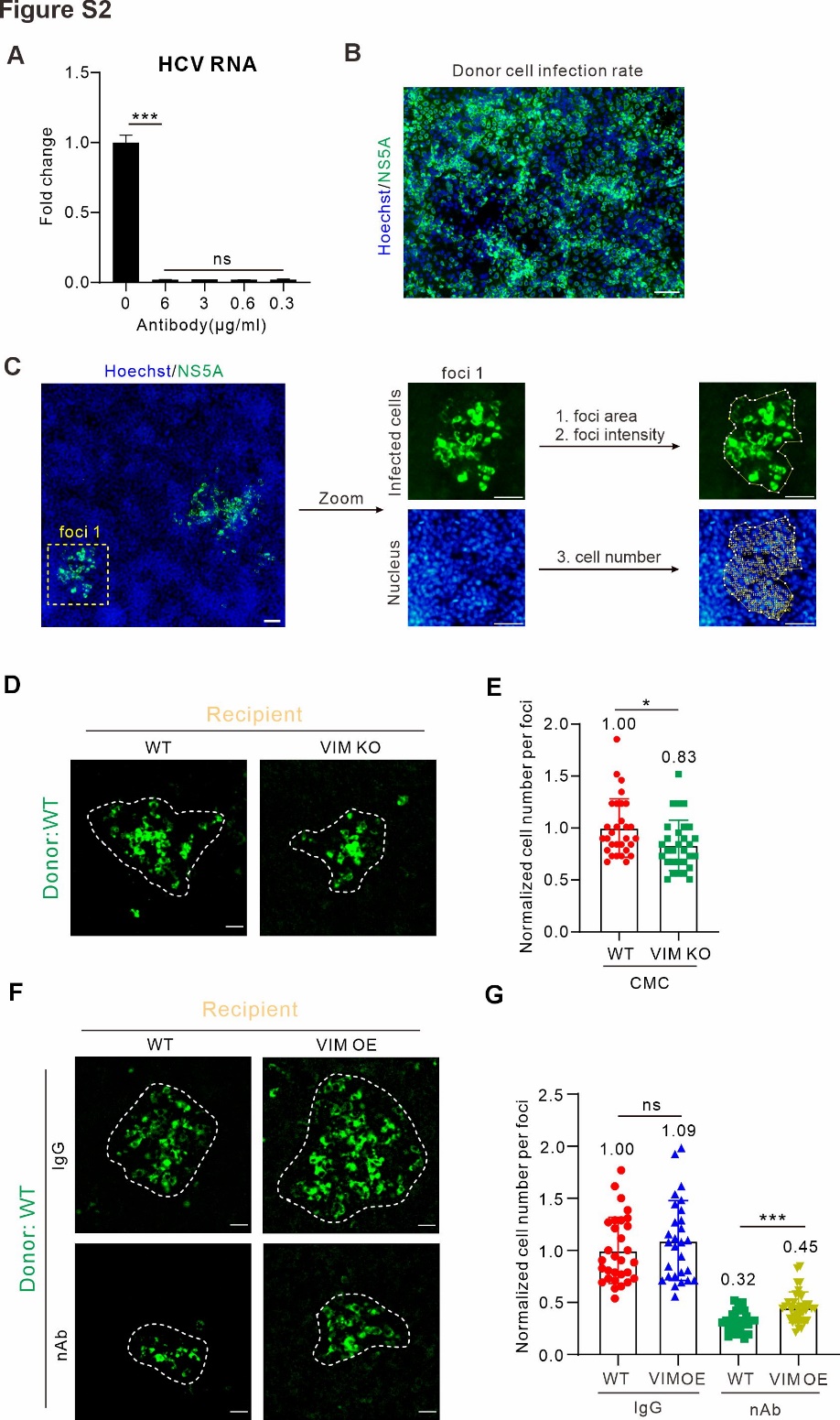


**Figure S2.** Vimentin is required for HCV cell-cell transmission. A) Cells were infected with HCV that pre-incubated with the antibody at 4°C for one hour at MOI of 1. HCV RNA were examined on 24 hours post infection. B) Verification of the infection rate of donor cells was conducted before co-cultivation. Blue channel represents cell nucleus and green channel represents HCV NS5A protein. Scale bars represent 100 µm. C) Statistical schematic of HCV cell-cell transmission foci. Blue channel represents cell nucleus and green channel represents HCV NS5A protein. Scale bars represent 50 µm. D) Representative fluorescent images of HCV-positive cell foci in ctrl and VIM KO cell lines in the condition of carboxymethylcellulose (CMC) sodium medium. Green channel represents HCV NS5A protein. Scale bars represent 50 µm. E) Quantification of HCV-positive cell number per foci in (D). n ≥ 25 foci were used for quantification in each group. F) Representative fluorescent images of HCV-positive cell foci in ctrl and VIM OE cell lines with / without HCV nAb. Green channel represents HCV NS5A protein. Scale bars represent 50 µm. G) Quantification of HCV-positive cell number per foci in (F). n≥25 foci were used for quantification in each group. Student *t* test was used for statistical analysis. No significant difference (ns), p > 0.05; ** p < 0.01; *** p < 0.001.


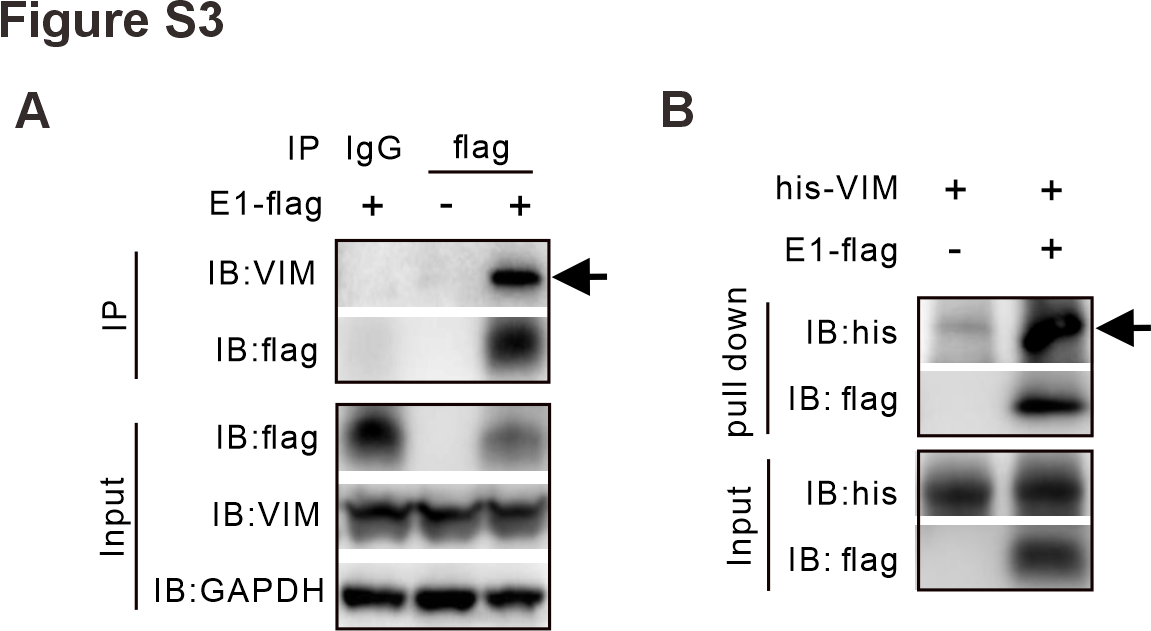


**Figure S3.** The interaction between E1 and vimentin. A) Interaction between flag-tagged E1 and endogenous vimentin. B) Pull-down assay followed by Western blotting to verify the interaction between flag-tagged E1 and purified His-vimentin protein.


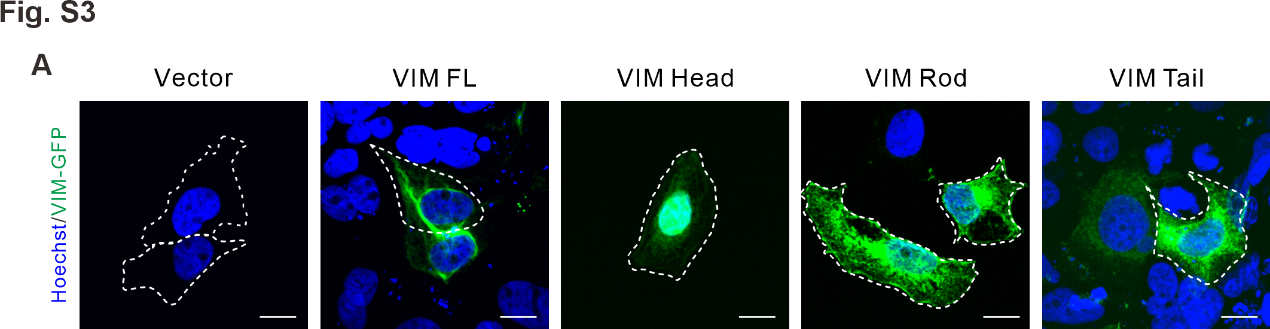


**Figure S4.** The cellular expression of different vimentin truncations. A) Representative immunofluorescence images of cells stained with Hoechst 33258 dye for nucleus (blue). Different truncations of vimentin-GFP plasmids were transfected into cells (green) to observe its intracellular localization. White dotted line represents the cell boundary. Scale bars represent 10 µm.


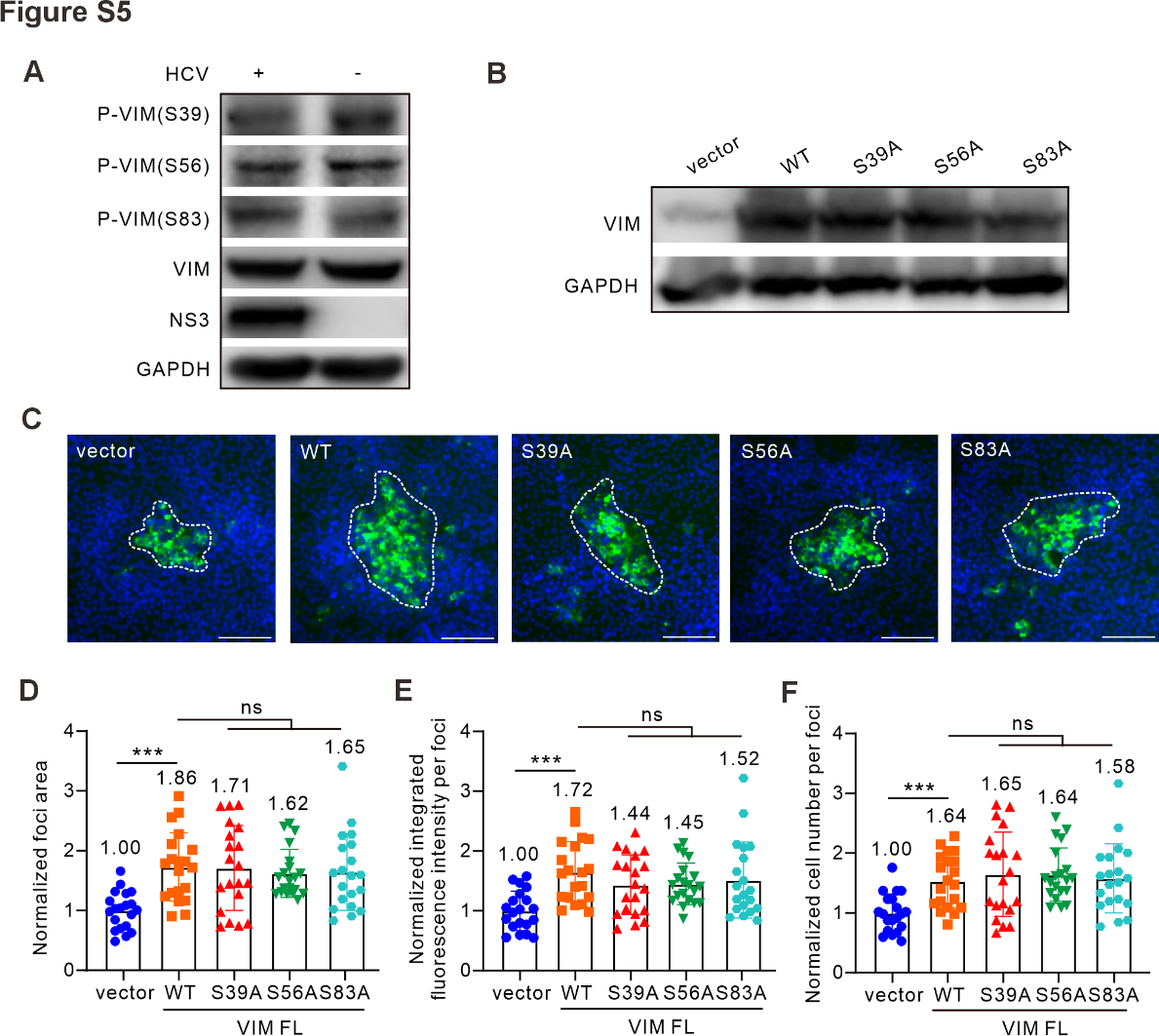


**Figure S5.** The effects of N-terminal phosphorylation on vimentin-mediated HCV cell-cell transmission. A) Western blotting verified the phosphorylation level of specific sites on vimentin after HCV infection (0.1 MOI, 72 hpi). B) Western blotting verified the overexpression of vimentin mutations. C) Representative fluorescent images of HCV-positive cell foci in different vimentin mutations overexpressed cell lines. Blue channel represents cell nucleus and green channel represents HCV NS5A protein. Scale bars represent 100 µm. D) Quantification of HCV-positive foci area in fluorescent images in (C). E) Quantification of HCV-positive foci integrated fluorescence intensity in fluorescent images in (C). F) Quantification of HCV-positive cell number per foci in fluorescent images in (C). n = 20 foci were used for quantification in each group. Data are represented as mean ± SD. Student *t* test was used for statistical analysis. No significant difference (ns), *** *p* < 0.001.
